# Worldwide productivity and research trend on fruit quality: a bibliometric study

**DOI:** 10.3389/fpls.2023.1294989

**Published:** 2024-01-09

**Authors:** Fei Ni, Ziwei Li, Jianzi Huang

**Affiliations:** ^1^ Guangdong Provincial Key Laboratory for Plant Epigenetics, College of Life Sciences and Oceanography, Shenzhen University, Shenzhen, China; ^2^ Key Laboratory of Optoelectronic Devices and Systems of Ministry of Education and Guangdong, College of Physics and Optoelectronic Engineering, Shenzhen University, Shenzhen, China; ^3^ College of Agriculture and Forestry Ecology, Shaoyang University, Shaoyang, China

**Keywords:** antioxidant, bibliometric analysis, fruit quality, hot topic, research trend

## Abstract

**Introduction:**

As one of the important sources of food for human beings, fruits have been extensively studied. To better guide basic and applied research, it is urgent to conduct a systematic analysis of these studies based on extensive literature collection.

**Methods:**

Based on the Web of Science Core Collection database, this study uses R language and CiteSpace to conduct bibliometric analysis and data mining on the literatures related to fruit quality from January 2013 to June 2023.

**Results:**

The results indicated that among various fruits, tomatoes have been most frequently studied with special interests in photosynthesis, fruit development, and molecular breeding. The research direction primarily focused on fruit resistance and storage characteristics. Among the indicators related to fruit quality, antioxidant activity has the highest co-occurrence with other indicators of fruit quality, especially with nutrients such as anthocyanins, phenolic substances, sugars, and fruit firmness.

**Discussion:**

Currently, adaptation to stress and antioxidant activity are recognized as prominent research focal points in this field. Fruit morphology, particularly fruit size, irrigation methods, application of molecular technology, and infection prevention, represent potential areas of interests in future research on fruit quality.

## Introduction

Fruit are essential sources of nutrition sources in people’s daily lives. Most kinds of fruits are rich in minerals and vitamins required by the human body (Martí et al., 2016; Vats et al., 2020), while certain fruits also contain various antioxidant active substances that are closely related to human health (Basu et al., 2018; Cámara et al., 2022). During the process of fruit development and ripening, there may be changes in color, aroma, flavor, and texture (Karlova et al., 2014). These changes are easily affected by nutritional status and environmental conditions of fruit crops (Chavarría-Perez et al., 2020).

Fruit quality includes both appearance quality and internal quality (Corpas and Palma, 2018). Appearance quality consists of fruit shape, fruit size, single fruit weight, and fruit color (Sofia and Sinéad, 2014; Martuscelli et al., 2016); while intrinsic quality mainly includes flavor, aroma, sugar acid ratio, soluble solids content, nutritional composition, and storage and transportation performance (Kucuker and Ozturk, 2014; Karakaya et al., 2020; Zhu et al., 2020; Faizy et al., 2021). The absolute content of sugars, amino acids, and vitamins in the fruit usually increases with fruit growth (Li et al., 2018), while the content of organic acids and pectin decreases with fruit maturity (Aubert et al., 2019). The sweetness mainly comes from the sugars in the fruit (Goldenberg et al., 2014). As fruit ripening, starch is rapidly converted into sucrose, glucose, and fructose, which make the fruit sweet (Etienne et al., 2013). The sour taste was mainly determined by the contents of organic acids in the fruits. During the process of fruit maturity, the organic acids were gradually decomposed into other organic substances (Beauvoit et al., 2018). The tannins in the fruit are gradually decomposed and converted into water-soluble substances, making the astringency of fruits weaken or disappear (Wu et al., 2022). Therefore, harvesting too early is unfavorable for improving fruit quality, whereas harvesting too late may affect the storage performance of fruits. To improve fruit quality, researches have been carried out on grafting (Fallik and Ziv, 2020), molecular breeding (Liao et al., 2021), cold storage (Karakaya et al., 2020; Islam et al., 2022), and new coatings (Lin et al., 2020).

During the last decades, there have been extensive studies on various aspects related to fruit quality (Musacchi and Serra, 2018; Zhu et al., 2018; Quinet et al., 2019; Ozturk et al., 2020; Liao et al., 2021; Mao et al., 2022; Ozturk et al., 2022; Pott et al., 2023). To provide better guidance for both basic and applied research, a systematic analysis of these studies based on extensive literature collection is urgently needed. Bibliometric analysis is a method for efficiently analyzing massive literature information (such as citation frequency, keywords, references, etc.) (Fu et al., 2013). It can construct a data matrix according to the similarity and measurement of information units, and then visually analyze the complex relationships between information units or groups such as networks and conceptual structures, thus providing objective and systematic analysis of the hot spots, dynamics, and development trends in the research field (Costa et al., 2019). This method has been widely used in summarizing researches on agronomy, environment, and other related fields (Yao et al., 2018; Kamdem et al., 2019; Zyoud and Fuchs-Hanusch, 2020).

Bibliometrics is the main quantitative evaluation method for analyzing the research hotspots and evolution of disciplines in recent years (Aria and Cuccurullo, 2018). It focuses on the literature system and bibliometric characteristics, uses statistical and mathematical methods to study the distribution structure, quantitative relationships, change patterns, and quantitative management of literature collections. By analyzing the quantitative characteristics of scientific research achievements in specific disciplinary fields, a discipline that explores the evolution characteristics of disciplines through the laws of change and their internal connections, and then explores certain structures, characteristics, and laws of scientific literature. This method can present bibliometric results through knowledge graphs and other means, which helps scholars understand the current research status and development process of specific disciplinary fields, clarify the evolution of scientific problems, and provide reference for future research directions.

In recent years, there has been relatively little quantitative analysis on fruit quality. It remains unclear what the recent research trends in fruit quality are, which areas are the focus, and what the future research hotspots will be. In this study, based on the Web of Science (WoS) Core Collection database, we take “fruit quality” as the theme word and use the Bibliometrix software package in R language to conduct bibliometric analysis of the literatures published from 2013 to 2023, with special attention on highly cited papers, keywords, and historical direct citations. By accurately searching and reading authoritative literature on fruit quality, this study summarizes the hot spots of previous researches on fruit quality and prospects the future research trends in this field. The objectives of this study include: (1) Revealing the fundamental patterns of fruit quality research, such as annual publication volume, research strength (countries, institutions, authors, journals), research hotspots, and topics; (2) Providing references and suggestions for future research on fruit quality from multiple perspectives, in order to analyze the research trends in the field of fruit quality and make significant contributions to the practice of breeding for fruit quality improvement.

## Materials and methods

### Data source and search strategy

Thomson Reuters’ Web of Science Network core set was selected as the database to obtain initial data (Mongeon and Paul-Hus, 2016). On June 31, 2023 the advanced search tool was used to search with the theme of “fruit quality” and the theme of TS = “fruit * quality”. All analyses were based on the original data exported at the time to exclude potential changes in retrieval records caused by re-retrieval. The retrieval set the document type as article, the language as English, and the document time span from 2013 to 2023. Unrelated research directions were removed from the research direction column. The full records of the obtained literature items and the cited references were exported for subsequent bibliometric analysis.

### Study selection and data management

In this study, only original articles published in English were included. Research that included the following content was excluded: (1) Summaries, conference abstracts, and conference papers; (2) Translated versions of articles or comments; (3) Comments, editorials, and letters; (4) Duplicate literatures. All information, including the number of papers and citations, title, author, affiliation, country, keywords, journal, publication year, and references, were collected. For the keywords with the same meaning but different expressions, we have processed them, leaving only one standardized keyword.

### Data processing

Bibliometric analysis was based on R language (Aria and Cuccurullo, 2018). The Bibliometrix software package (version 4.1.0) was used to draw the knowledge domain map of author contribution and collaboration, journal co-citation and keyword timeline view, and keyword co-occurrence. The reference context was drawn using CiteSpace (version 5.7.r5) (Chen and Leydesdorff, 2013).

The CiteSpace parameters were as follows: link retaining factor (LRF = 3), look back years (LBY = -1), e for top N (e = 2), time span (2013 - 2023), years per slice (1), links (strength: cosine, scope: within slices), selection criteria (g-index: k = 25), and minimum duration (MD = 5).

## Results

### General description

**Figure 1:**
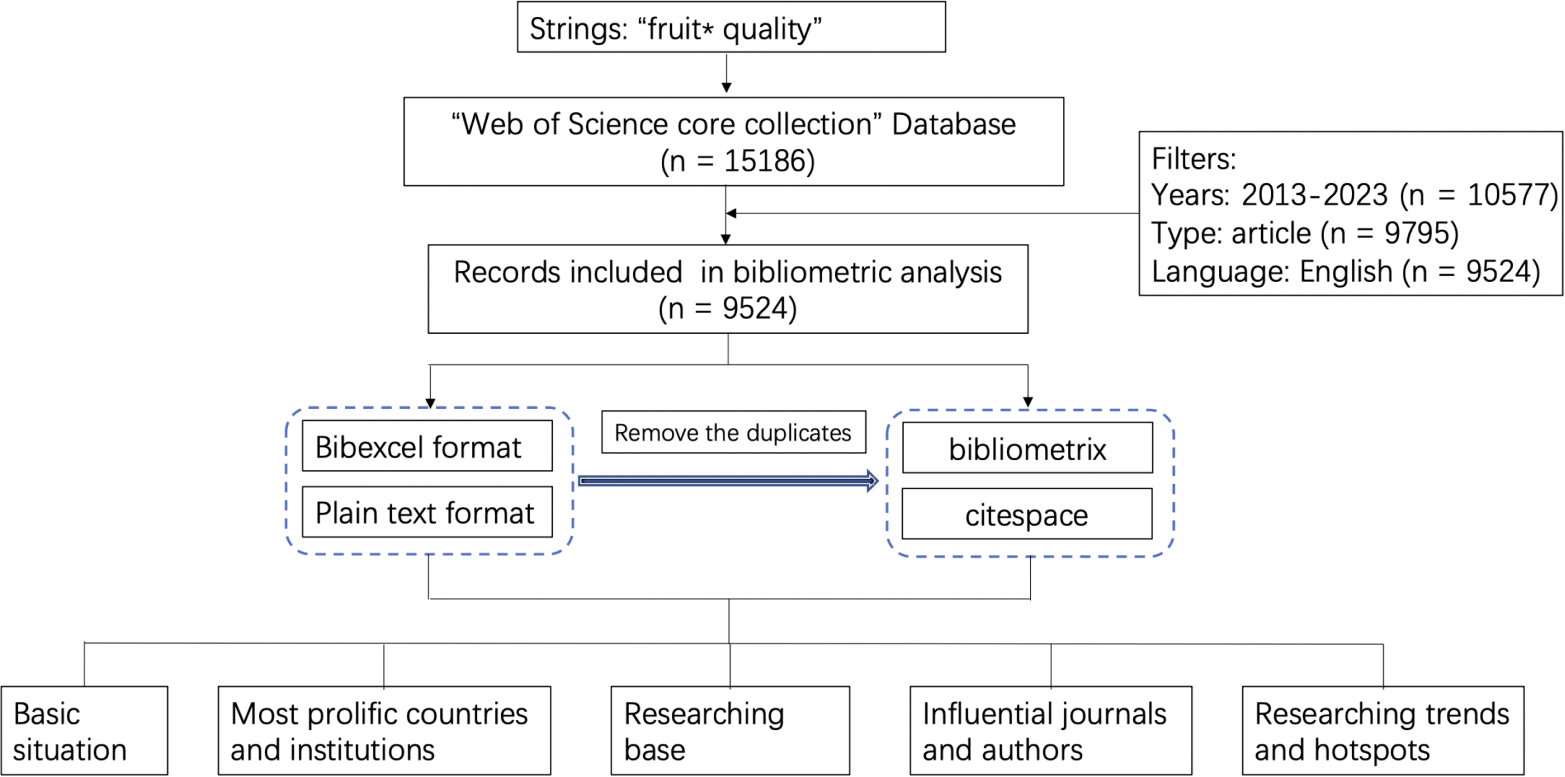
The data collecting and filtering process for the bibliometric analysis of fruit quality.

A total of 15186 publications with the theme of “fruit * quality” were retrieved from the WoS Core Collection database. Among these publications, 10577 were published between January 2013 and June 2023 Subsequently, reviews and conference abstracts (n=782) as well as non-English publications (n=271) were screened out. Finally, 9524 articles were obtained for bibliometric analysis (**Figure 1**).

The annual total number of articles reporting researches on fruit quality showed an overall growth trend over the past decade (**Supplementary Table 1**). From 2013 to 2017, the annual increase in the total number of articles was less than 10%. Since 2018, the number of articles grew more rapidly and surged for the first time between 2019 and 2021. It was worth noting that the proportion of fruit-quality-related articles reached 17% in 2021. The research on fruit quality is still in the stage of rapid development. The average number of citations per article per year also showed a growth trend (**Supplementary Table 1**). Since 2013, it remained above 2.5, reaching the highest of 3 in 2019. The research on fruit quality gains increasing attention in the past decade.

### Countries and districts

**Figure 2:**
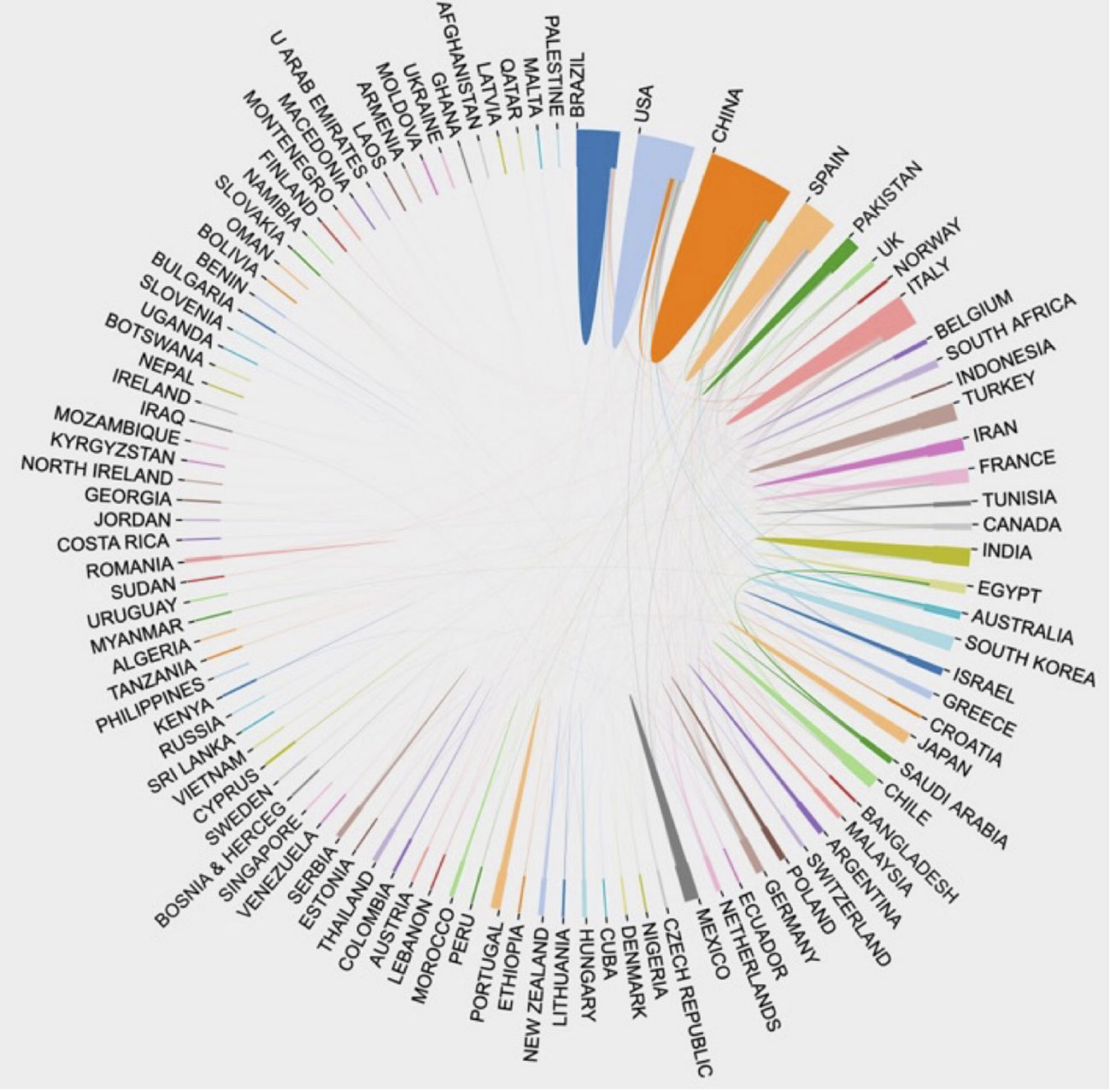
Diagram of collaboration between countries.

A total of 100 countries worldwide have contributed to the publications on fruit quality. There were 23 countries with total citations above 1000 (**Supplementary Table 2**). China (31076/104387, 29.8%) had the highest number of total citations, accounting for more than 25% of all citations. Spain (11319/104387, 10.81%) and the United States (11304/104387, 10.83%) ranked the second and the third place, respectively. Although the numbers of total citations for Singapore, Lebanon, Denmark, Sweden, and Netherlands were not as large as those for the top 3 countries, the average numbers of citations per article for these countries were more than those for most countries, with the value for Singapore being as high as 45.7 (**Supplementary Table 3**), indicating that the quality of fruit-quality-related research carried out in these countries was relatively high. Close research cooperation among countries might promote research progress. For instance, China had the closest cooperation with the United States in the field of fruit quality (**Figure 2**).

### Institutions

Many universities or institutions have significantly contributed to the study of fruit quality. Extensive cooperative network analysis was carried out among these universities or institutions. In the network diagram of the top 48 institutions, 11 clusters were formed (**Supplementary Figure 1**). Washington State University and University of Florida in the United States, China Agricultural University and Northwest Agriculture and Forestry University in China have made important contributions to most publications in the world. As for international cooperation, Washington State University and University of Florida cooperate closely with other research institutions in the United States, while Cornell University cooperates closely with many Chinese research institutions.

### Journals

This study showed that a total of 725 journals published articles on fruit quality research and listed the top 20 most productive journals (**Supplementary Table 4**). The total citation (TC) and H-index of “Scientia Horticulturae” ranked the highest. “Food Chemistry” and “Postharvest Biology and Technology” ranked the second and the third place, with H-index of 45 and TC of 5454 and 8589, respectively. Among them, “Postharvest Biology and Technology” had the highest total number of articles.

### References

Dual map was used to describe the reference relationship (**Supplementary Figure 2**). Each spline starts with the reference map on the left and points to the reference map on the right. In the current map, three main reference paths were displayed. Published articles were mainly published in journals within the fields of ‘Molecular’ and ‘Biological Sciences’, while most of the cited articles were published in the fields of ‘Plant’, ‘Ecology’; ‘Environment’, ‘Nutrition’; ‘Molecular’, ‘Biology’, ‘Genetics’.

### Analysis of keywords

**Figure 3:**
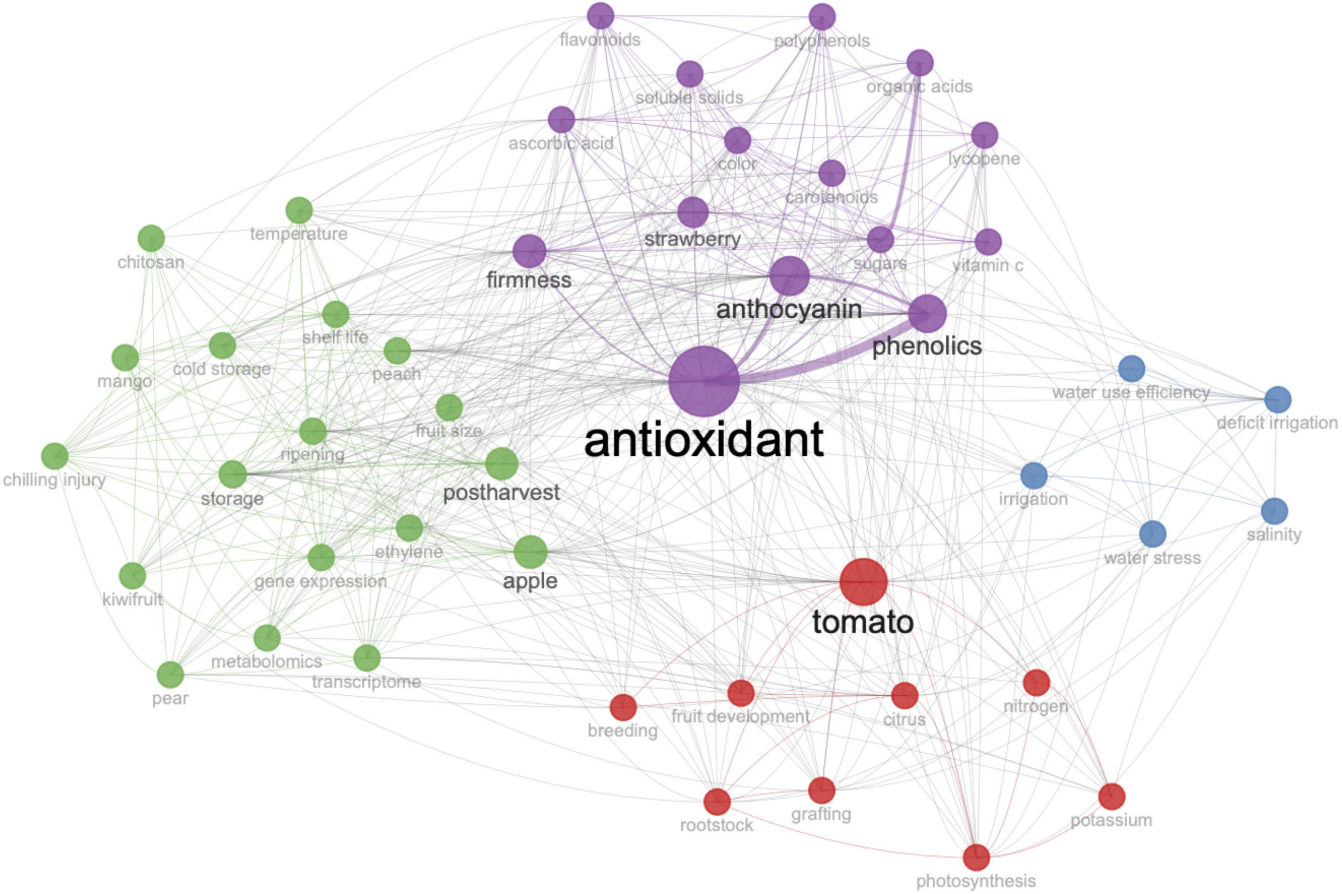
Keywords co-occurrence network of researches on fruit quality from 2013 to 2023.

In the keywords co-occurrence network map of fruit-quality-related articles from 2013 to 2023, the keywords were divided into four groups (**Figure 3**). The keyword nodes in these four groups were ‘postharvest’, ‘tomoto’, ‘antioxidant’ and ‘irrigation’. Group 1 (green) indicated that the storage characteristics of fruits played an important role in fruit quality, and the researches in this group mainly focused on the morphological and physiological changes of fruit quality after harvest. Group 2 (red) implied that the most frequently used plant species for study on fruit quality were tomato and citrus, and the researches in this group paid special attention on the effects of plant growth state, grafting, photosynthesis, and salt stress on the fruit quality of tomato and citrus. Group 3 (purple) focused on sugars and the substances with antioxidant activity including anthocyanins, firmness, phenolics, and carotenoids, and there were many related studies in strawberry. Group 4 (blue) mainly consisted of researches on the impact of water use on fruit quality.

**Figure 4:**
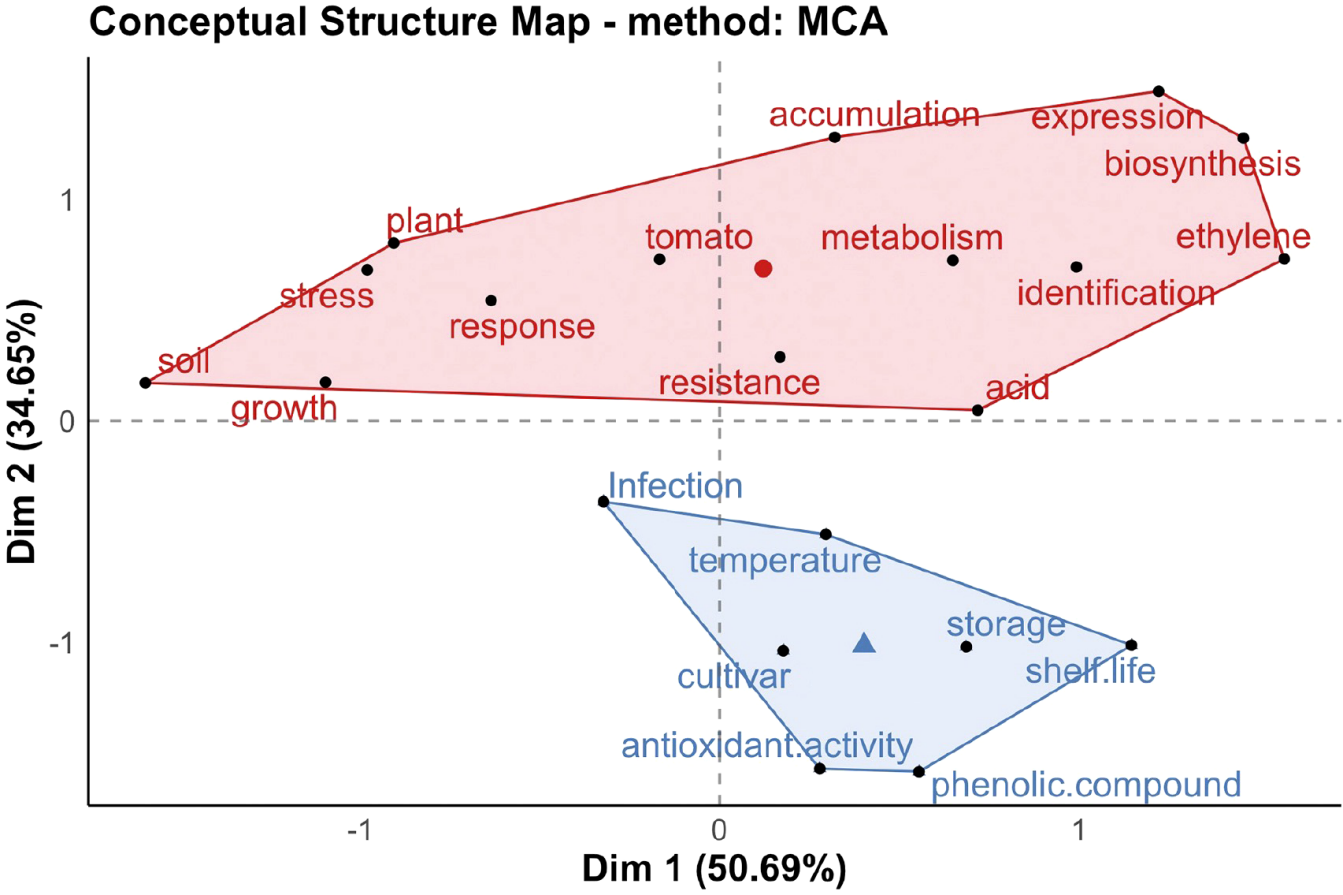
Multiple correspondence analysis of researches on fruit quality from 2013 to 2023.

**Figure 5:**
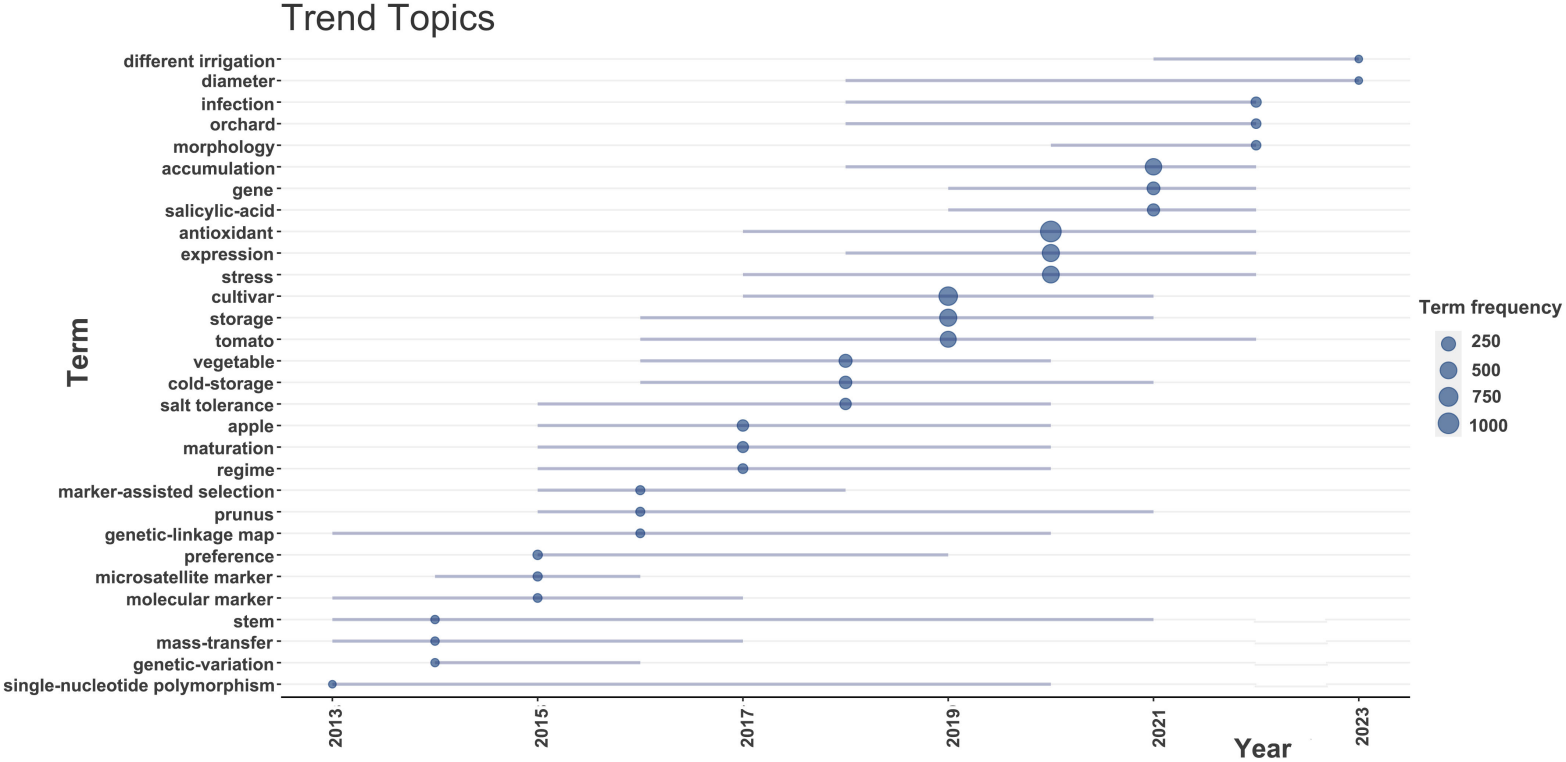
Trend topics of researches on fruit quality from 2013 to 2023.

The multiple correspondence analysis divided the fruit-quality-related researches into two categories (**Figure 4**). Axis 1 (dim 1) and axis 2 (dim 2) explained 50.69% and 34.65% of the total variance, respectively, with a cumulative interpretation rate of 85%. There were significant differences between different clusters. In cluster 1 (red region), most keywords focused on the effects of adversity on fruit quality, the characteristics of material synthesis and accumulation in relation to fruit quality, and the expression of related genes. In cluster 2 (blue region), the keywords mainly included storage, temperature, shelf life, antioxidant activity, cultivars, and phenolic compounds, which indicated that these studies were more interested in the post-harvest storage status of fruits. Next, we identified the research trends in the field of fruit quality, to provide reference for future researchers. We analyzed and visualized the time trend of keywords (**Figure 5**). In the last decade, new trends in fruit quality research included different irrigation and diameter.

## Discussion

In this study, we conducted a bibliometric analysis of the literatures related to fruit quality research in the past decade to summarize the key hot spots and trends in this research field. We found that the number of literatures related to fruit quality increased rapidly since 2013, especially after 2019. In 2013, there were two landmark articles, one on genomic selection of apple fruit traits (Kumar et al., 2012) and the other on the composition of sugars, organic acids, and total phenolics in berries (Mikulic-Petkovsek et al., 2012), which laid a foundation for the subsequent research on apple and berry fruit quality. Subsequently, the influence of chitosan on fruit quality became research hotspots in several species, such as strawberry (*Fragaria ananassa*) and guava (*Psidium guajava*) (Wang and Gao, 2013; Nair et al., 2018). The research on the fruit quality of sweet cherry and its correlation with human health also received extensive attention (Ballistreri et al., 2013; Correia et al., 2017). In addition, the effect of foliar fertilizer and xylem formation on fruit quality has gradually become a research hotspot in recent years.

The overall retrieved literatures in relation to fruit quality during the past decade were published in 725 journals, with a total of 9524 articles. The annual growth rate was 10.6%, while the average number of citations per article was 13. An average of 845.4 articles were published every year. Since 2018, the number of articles published every year has increased significantly.

More than 50% of the fruit-quality-related publications were contributed by the researchers from China, Spain, and the United States, suggesting that the researchers from these three countries played an important role in publication output and general citations. The high ranking of Singapore, Lebanon, and Denmark in terms of total citations showed the high quality of research activities in these countries. In terms of international cooperation, the United States and China had close cooperation with other countries, while Singapore had a relatively low degree of cooperation with other countries. Our analysis also showed that the journals with great influence on fruit quality were Scientia Horticulturae, Postharvest Biology and Technology, and Food Chemistry, and the TC and H-index of Scientia Horticulturae were the highest.

According to the keyword co-occurrence map and multiple correspondence analysis, the researches on fruit quality mainly focused on storage quality, nutritional quality, antioxidant activity, water use, and adversity impact. There were many studies on the fruits of tomato, citrus, apple, and blueberry. Visual analysis of the occurrence of keywords showed the new trends in fruit quality research mainly included xylem and trifoliate orange, which provided potential directions for the future research.

For the first time, our work used CiteSpace and R language to conduct bibliometric analysis on fruit quality research. This work based on the WoS core collection, which was the most important data source for quantitative analysis of scientific literatures (Chen et al., 2015). In addition to analyzing the social network between countries or institutions, we also performed keyword clustering analysis, which clearly showed the research hotspots and the development trends in this field (Li et al., 2016).

With the improvement of living standards, people’s demand for fruit quality is also increasing. In the past 10 years, researchers have been committed to improving fruit quality, including environmental impact research, nutritional research, biological research, gene mining, etc. We selected publications related to fruit quality research in the past 10 years, analyzed the development status of research in this field, and summarized the following three research directions with development potential for the future work.

Firstly, fruit appearance is an important aspect of fruit quality, which affects consumer preferences and market sales. The attractiveness of fruit appearance depends on its size, shape, color, texture, and surface features. Fruit size is a key factor that affects consumer recognition and acceptance, as larger fruit is usually associated with higher quality and greater value. Fruit shape also plays a crucial role in consumer preferences, and unique shapes are often used to distinguish different cultivars or variants. Research can be conducted from genomics, growth regulation mechanisms, genetic improvement, environmental factors, etc.

Secondly, irrigation has an important impact on fruit quality. By reasonable irrigation management, fruit quality can be improved, and fruit yield and quality can be ensured. By studying the advantages and disadvantages of irrigation water treatment technology, different irrigation methods, different irrigation timing, and different irrigation amounts, suitable methods can be selected to improve fruit quality. In the future, further research can be conducted on the relationship between irrigation and fruit nutrient components, such as water and nutrient elements, and their impact on fruit nutrient components. By studying the relationship between irrigation and fruit nutrient components, the most suitable irrigation management strategy can be selected to improve fruit quality.

Thirdly, pathogen infection is an important factor affecting fruit quality. It is a very important task to reduce the impact of diseases on fruit quality by improving disease prevention and control technology. In the future, further research can be conducted on the impact mechanism of different diseases on fruit nutrient components, storage period, and appearance quality, and explore suitable prevention and control strategies, such as chemical prevention and control, biological prevention and control, physical prevention and control, etc., to improve fruit quality.

Our research also has some limitations. Firstly, we only include articles on fruit quality published in the WoS database. Although WoS is considered the most important data source for scientific bibliometric analysis (Chen et al., 2015), it may not fully reflect the status of all researches on fruit quality. Secondly, almost all included studies are in English, which may lead to selection bias. Therefore, the results may not be applicable to fruit quality published in other languages (Li et al., 2016). Thirdly, there are some inconsistencies in the data analysis process, such as an author from different units, an organization with different names, and keywords with the same meaning having different expression. Although we standardize institutions and keywords in our research, potential errors may still exist.

## Conclusions

Through bibliometric analysis, we have identified promising research prospects in the field of fruit quality. Publications related to fruit quality are experiencing rapid growth. Currently, adaptation to stress and antioxidant activity are recognized as prominent research focal points within this domain. Fruit morphology, particularly fruit size, irrigation methods, application of molecular technology and infection prevention, represent potential areas of interest in fruit quality research. It is important to highlight that research on fruit quality predominantly centers around tomatoes and apples, with comparatively limited exploration of other fruit crop species. Addressing this research gap necessitates further investigation to comprehensively cover the spectrum of fruit quality attributes.

## Data availability statement

The original contributions presented in the study are included in the article/**Supplementary Material**. Further inquiries can be directed to the corresponding author.

## Author contributions

FN: Writing – original draft. ZL: Writing – review & editing. JH: Writing – review & editing.

## Supplementary material

Supplementary Figure 1 Extensive cooperative network analysis of universities and institutions.

Supplementary Figure 2 Dual map: reference relationship context. Yellow, green, and purple splines represent references to source articles.
