# Supplementary figures and images for: Worldwide productivity and research trend on fruit quality: a bibliometric study
Source: Front Plant Sci. 2024 Jan 9;14:1294989. doi: 10.3389/fpls.2023.1294989 (PMC10803653; doi:10.3389/fpls.2023.1294989)

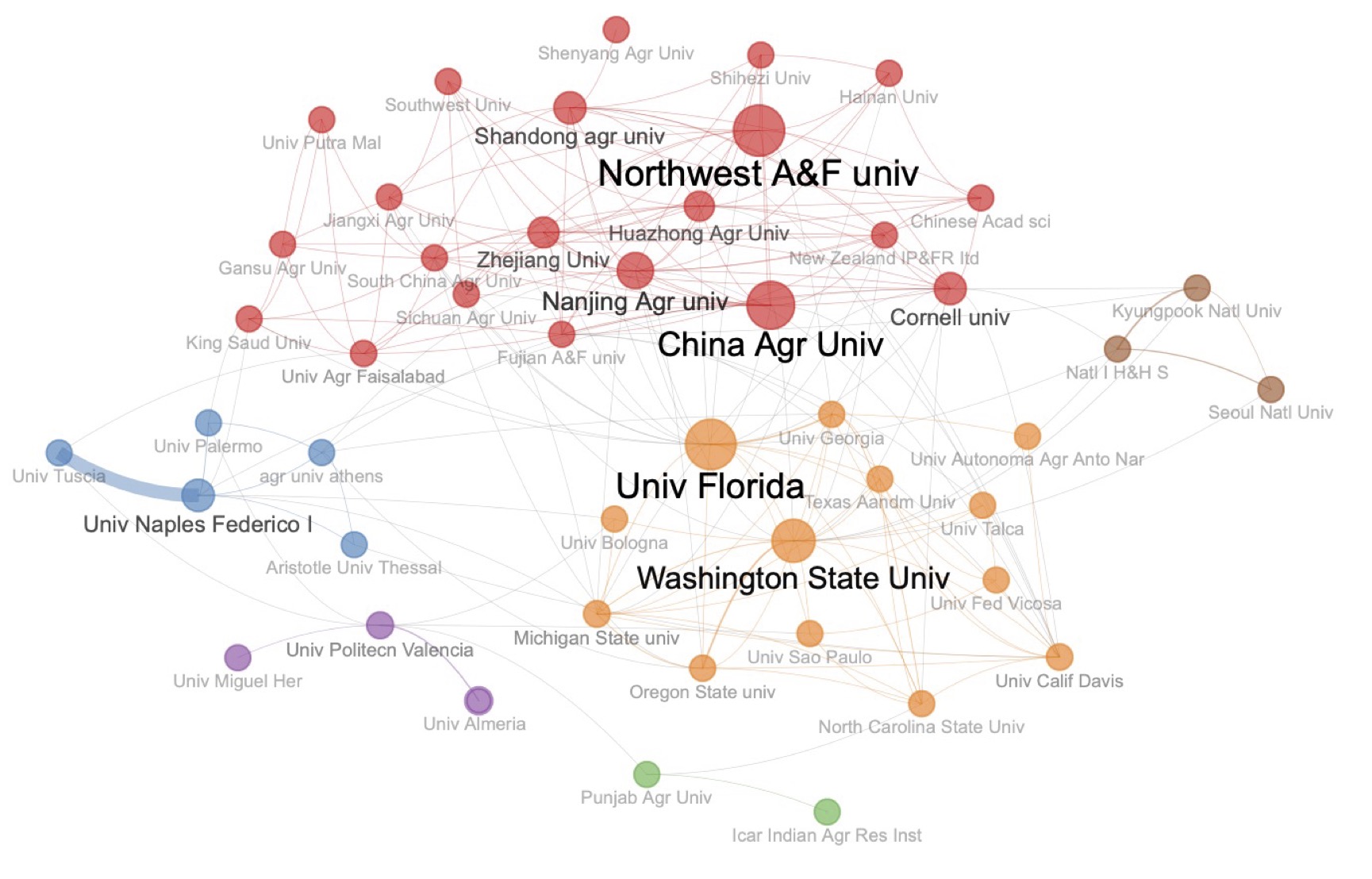

Supplement: Supplementary Figure 1 — Extensive cooperative network analysis of universities and institutions. [file Image_1.jpeg]

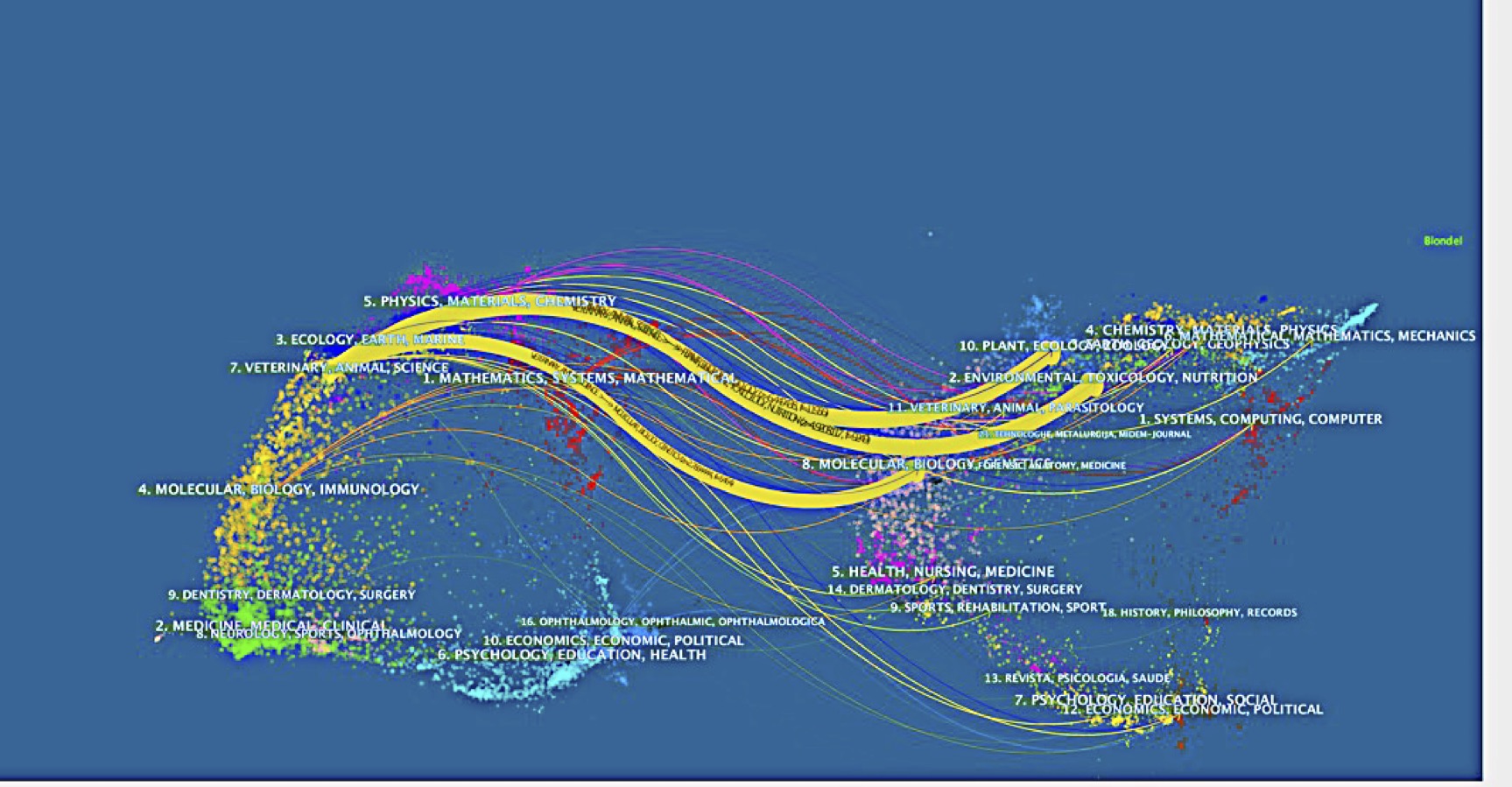

Supplement: Supplementary Figure 2 — Dual map: reference relationship context. Yellow, green, and purple splines represent references to source articles. [file Image_2.jpeg]
