# Supplementary material for: Worldwide productivity and research trend on fruit quality: a bibliometric study
Source: Front Plant Sci. 2024 Jan 9;14:1294989. doi: 10.3389/fpls.2023.1294989 (PMC10803653; doi:10.3389/fpls.2023.1294989)
Supplement: Supplementary file 3 [file Table_1.docx]

| Year | Articles | Mean TC per Year | Mean TC per Article |
| --- | --- | --- | --- |
| 2013 | 456 | 2.59 | 28.5 |
| 2014 | 498 | 2.69 | 26.87 |
| 2015 | 573 | 2.60 | 23.41 |
| 2016 | 613 | 2.63 | 21.08 |
| 2017 | 718 | 2.77 | 19.36 |
| 2018 | 758 | 2.70 | 16.17 |
| 2019 | 934 | 3.00 | 14.94 |
| 2020 | 1066 | 2.90 | 11.58 |
| 2021 | 1325 | 2.51 | 7.52 |
| 2022 | 1513 | 1.44 | 2.87 |
| 2023 | 1070 | 0.56 | 0.56 |

Supplementary table 1 Annual publications and the average article citations for the articles related to fruit quality for the articles related to fruit quality

Supplementary table 2 Top 23 Total citations and average citations per article of the fruit-quality-related articles from different countries.

| Countries | Total Citations | Average Article Citations |  |
| --- | --- | --- | --- |
| CHINA | 31076 | 13.30 |  |
| SPAIN | 11319 | 17.10 |  |
| USA | 11304 | 13.10 |  |
| ITALY | 11285 | 20.40 |  |
| BRAZIL | 4669 | 7.60 |  |
| IRAN | 4078 | 14.10 |  |
| INDIA | 3881 | 7.70 |  |
| TURKEY | 2657 | 7.40 |  |
| FRANCE | 2544 | 23.80 |  |
| GERMANY | 2210 | 17.50 |  |
| CHILE | 1779 | 12.10 |  |
| KOREA | 1660 | 7.90 |  |
| MEXICO | 1651 | 9.00 |  |
| ISRAEL | 1626 | 20.10 | |
| SOUTH AFRICA | 1551 | 15.80 | |
| JAPAN | 1495 | 9.10 | |
| EGYPT | 1483 | 10.90 | |
| PORTUGAL | 1440 | 16.60 | |
| POLAND | 1434 | 8.30 | |
| PAKISTAN | 1384 | 7.70 | |
| AUSTRALIA | 1347 | 11.50 | |
| CANADA | 1299 | 10.90 | |
| BELGIUM | 1215 | 28.30 | |

Supplementary table 3 Average article citations and total citations countries concerning fruit quality

| Country | Average Article Citations |  | TC |
| --- | --- | --- | --- |
| SINGAPORE | 45.67 |  | 137 |
| LEBANON | 29.75 |  | 119 |
| DENMARK | 26.33 |  | 395 |
| SWEDEN | 26.13 |  | 209 |
| NETHERLANDS | 26.11 |  | 940 |
| SYRIA | 25.00 |  | 25 |
| BELGIUM | 24.51 |  | 1054 |
| SWITZERLAND | 23.58 |  | 731 |
| FRANCE | 20.21 |  | 2162 |
| NEW ZEALAND | 19.91 |  | 1055 |
| UNITED KINGDOM | 19.85 |  | 655 |
| ITALY | 18.82 |  | 9730 |
| FINLAND | 18.00 |  | 54 |
| SPAIN | 16.88 |  | 10666 |
| SLOVENIA | 16.33 |  | 784 |
| GERMANY | 15.70 |  | 1947 |
| ISRAEL | 15.61 |  | 1280 |
| SOUTH AFRICA | 15.08 |  | 1372 |
| DOMINICA | 15.00 |  | 15 |
| SRI LANKA | 14.83 |  | 89 |
| PORTUGAL | 14.05 |  | 1068 |
| CYPRUS | 13.71 |  | 425 |
| BOSNIA | 13.00 |  | 117 |
| MALAYSIA | 12.88 |  | 773 |
| ECUADOR | 12.80 |  | 64 |

Supplementary table 4 Top 20 most productive and impact journals concerning fruit quality field.

|  | Element | h-index | TC |
| --- | --- | --- | --- |
| 1 | SCIENTIA HORTICULTURAE | 49 | 14950 |
| 2 | FOOD CHEMISTRY | 45 | 5454 |
| 3 | POSTHARVEST BIOLOGY AND TECHNOLOGY | 45 | 8589 |
| 4 | AGRICULTURAL WATER MANAGEMENT | 36 | 4671 |
| 5 | FRONTIERS IN PLANT SCIENCE | 34 | 4055 |
| 6 | JOURNAL OF THE SCIENCE OF FOOD AND AGRICULTURE | 30 | 3161 |
| 7 | HORTSCIENCE | 27 | 3071 |
| 8 | PLOS ONE | 27 | 2003 |
| 9 | JOURNAL OF AGRICULTURAL AND FOOD CHEMISTRY | 24 | 1499 |
| 10 | AGRONOMY-BASEL | 23 | 2714 |
| 11 | LWT-FOOD SCIENCE AND TECHNOLOGY | 23 | 1585 |
| 12 | BMC GENOMICS | 22 | 1061 |
| 13 | HORTICULTURE RESEARCH | 22 | 1478 |
| 14 | BMC PLANT BIOLOGY | 21 | 1295 |
| 15 | JOURNAL OF EXPERIMENTAL BOTANY | 21 | 1326 |
| 16 | PLANT PHYSIOLOGY AND BIOCHEMISTRY | 20 | 1293 |
| 17 | TREE GENETICS \& GENOMES | 19 | 858 |
| 18 | MOLECULES | 18 | 841 |
| 19 | FOOD AND BIOPROCESS TECHNOLOGY | 17 | 943 |
| 20 | JOURNAL OF FOOD ENGINEERING | 17 | 761 |
